# Empathy as a predictor of burnout syndrome in health professionals of the Colombian Caribbean

**DOI:** 10.1192/j.eurpsy.2024.1230

**Published:** 2024-08-27

**Authors:** E. P. Ruiz Gonzalez, M. N. Muñoz Argel, A. M. M. Romero Otalvaro, M. G. Garcia Castañeda, M. C. Crespi

**Affiliations:** ^1^ Universidad Pontificia Bolivariana; ^2^Universidad de Cordoba, Montería, Colombia; ^3^Universidad de Buenos Aires, Buenos Aires, Argentina

## Abstract

**Introduction:**

Empathy is an essential skill in the doctor-patient relationship since it contributes to improve aspects of health care and patient satisfaction. Nevertheless, burnout research projects have been developed in recent years.

**Objectives:**

To examine the predictive capacity that empathy has on burnout syndrome in health professionals.

**Methods:**

A non-experimental, cross-sectional design was proposed. The type of study was correlational-descriptive since it was sought out to explore a functional relation through the prognosis of a criterion variable. Sample: 200 (100 female and 100 male).

**Results:**

First, the variance of cognitive and Affective Empathy was dug out in the emotional exhaustation criterion scale. Results accounted for 15% of variability in emotional exhaustation. (Corrected R 2 = .15, F = 17,56, p = 0,00). The best predictor of emotional exhaustation refers to Cognitive Empathy. (B = -.27, p = 0.00). It does not seem that Affective Empathy acts as a predictor variable of Emotional Exhaustation. (Table 1).

**Table 1:** Multiple linear regression analysis considering Emotional Exhaustation as a criterion.

TECA	*Corrected R*^2^	F	B	p
Cognitive Empathy	.15	17,5	-.27**	0,00
Affective Empathy			-.14	.13

The predictive capacity of Empathy in relation to Depersonalization was estimated (Corrected R 2 = .20, F = 25,4, p = 0.00). Cognitive and affective empathy were included as predictor variables and MBI as a criterion variable (Table 2). On one hand, the best predictor of Depersonalization is the Cognitive Empathy. On the other hand, regarding Affective Empathy, it does not act as a predictor of Depersonalization.

**Table 2:** Multiple linear regression analysis considering Depersonalization as a criterion.

TECA	*Corrected R*^2^	F	B	p
Cognitive Empathy	.20	25,4	-.32**	0,00
Affective Empathy			-.15	.84

Lastly, the predictive capacity of Empathy in relation to Personal Achievement was figured out. (Corrected R 2 = .19, F = 23,4, p = 0.00). Cognitive Empathy is the best predictor for Personal Fulfillment (Table 3).

**Table 3:** Multiple linear regression analysis considering Personal Fullfilment as a criterion.

TECA	*Corrected* R^2^	F	β	p
Cognitive Empathy	.20	25,4	.43**	0,00
Affective Empathy			.00	.96

**Conclusions:**

It was noticed that through a linear multiple regression analysis, the variable that best explains Emotional Exhaustation is Cognitive Empathy. Those results are replicated for Depersonalization and Personal Fullfilment.

**Disclosure of Interest:**

None Declared

